# The bionomics of the malaria vector *Anopheles rufipes* Gough, 1910 and its susceptibility to deltamethrin insecticide in North Cameroon

**DOI:** 10.1186/s13071-018-2809-5

**Published:** 2018-04-18

**Authors:** Parfait H. Awono-Ambene, Josiane Etang, Christophe Antonio-Nkondjio, Cyrille Ndo, Wolfgang Ekoko Eyisap, Michael C. Piameu, Elysée S. Mandeng, Ranaise L. Mbakop, Jean Claude Toto, Salomon Patchoke, Abraham P. Mnzava, Tessa B. Knox, Martin Donnelly, Etienne Fondjo, Jude D. Bigoga

**Affiliations:** 1 Research Institute of Yaounde (IRY), Organization de Coordination pour la lutte contre les Endémies en Afrique Centrale (OCEAC), B.P. 288, Yaoundé, Cameroon; 2 Department of Biological Sciences, Faculty of Medicine and Pharmaceutical Sciences, University of Douala, P.O. Box 2701, Douala, Cameroon; 3 Faculty of Sciences, University of Douala, P.O. Box 2701, Douala, Cameroon; 4 Ecole des Sciences de la Santé, Université Catholique d’Afrique Centrale, B.P. 1110, Yaoundé, Cameroon; 5 Faculty of Sciences, University of Yaoundé I, P.O. Box 812, Yaounde, Cameroon; 6 National Malaria Control Programme, Ministry of Public Health, P.O. Box 14386, Yaoundé, Cameroon; 7 The African Leaders Malaria Alliance (ALMA), 3 Barack Obama Drive, P.O. Box 70198, 11101 Dar es Salaam, Tanzania; 8 Global Malaria Programme, World Health Organization, Avenue Appia, Geneva, Switzerland; 9 Department of Vector Biology, Liverpool School of Tropical Medicine, Pembroke Place, Liverpool, L3 5QA UK; 10 National Reference Unit for Vector Control, The Biotechnology Center, University of Yaoundé I, P.O. Box 3851-Messa, Yaoundé, Cameroon

**Keywords:** Malaria vector, *Anopheles rufipes*, Bionomics, Deltamethrin susceptibility

## Abstract

**Background:**

Following the recent discovery of the role of *Anopheles rufipes* Gough, 1910 in human malaria transmission in the northern savannah of Cameroon, we report here additional information on its feeding and resting habits and its susceptibility to the pyrethroid insecticide deltamethrin.

**Methods:**

From 2011 to 2015, mosquito samples were collected in 38 locations across Garoua, Mayo Oulo and Pitoa health districts in North Cameroon. Adult anophelines collected using outdoor clay pots, window exit traps and indoor spray catches were checked for feeding status, blood meal origin and *Plasmodium* circumsporozoite protein. The susceptibility of field-collected *An. rufipes* to deltamethrin was assessed using WHO standard procedures.

**Results:**

Of 9327 adult *Anopheles* collected in the 38 study sites, *An. rufipes* (6.5%) was overall the fifth most abundant malaria vector species following *An. arabiensis* (52.4%), *An. funestus* (*s.l.*) (20.8%), *An. coluzzii* (12.6%) and *An. gambiae* (6.8%). This species was found outdoors (51.2%) or entering houses (48.8%) in 35 suburban and rural locations, together with main vector species. Apart from human blood with index of 37%, *An. rufipes* also fed on animals including cows (52%), sheep (49%), pigs (16%), chickens (2%) and horses (1%). The overall parasite infection rate of this species was 0.4% based on the detection of *P. falciparum* circumsporozoite proteins in two of 517 specimens tested. Among the 21 *An. rufipes* populations assessed for deltamethrin susceptibility, seven populations were classified as “susceptible” (mortality ≥ 98%) , ten as “probable resistant” with a mortality range of 90–97% and four as “resistant” with a mortality range of 80–89%.

**Conclusions:**

This study revealed changeable resting and feeding behaviour of *An. rufipes*, as well as further evidence on its ability to carry human malaria parasites in North Cameroon. Besides, this species is developing physiological resistance to deltamethrin insecticide which is used in treated nets and agriculture throughout the country, and should be regarded as one of potential targets for the control of residual malaria parasite transmission in Africa.

## Background

The *Anopheles* fauna of the Afrotropical region has about 150 species, and almost 20 species are involved in the transmission of malaria parasites to humans [1]. Between 2010 and 2015, there was a 13% reduction of the population at risk of malaria in sub-Saharan Africa. However, most cases (90%) and deaths (92%) still occur in the WHO African Region [2]. In Cameroon, the disease is endemic throughout the country, with some variations on the transmission intensity in specific areas such as highlands and Sahel. The estimated number of malaria cases in the country was almost 1.2 million cases in 2013 [3]. The vast majority of cases and related deaths are due to *P. falciparum*, with *P. malariae* and *P. ovale* species being of minor importance. *Plasmodium falciparum* malaria is responsible for 36% of outpatient consultation, 67% of childhood mortality and 48% of hospital admissions [4]. Various interventions such as targeted case management of vulnerable groups (children under 5) and mass prevention strategies mostly based on the general use of long-lasting insecticidal nets (LLINs) against vectors have contributed to significantly reduce the overall malaria prevalence from 46.3% in 2008 to 26.5% in 2013 [4]. However, this progress may be compromised by the risk of development of drug resistance in parasites and of vector resistance to insecticides.

Vector control is currently a key strategy to prevent malaria in Cameroon and other endemic countries. Six anopheline species also distributed across the African region are considered as main local vectors of human malaria parasites: *Anopheles gambiae* Giles, 1902; *An. coluzzii* Coetzee et al., 2013 (see [5]); *An. funestus* Giles, 1900; *An. arabiensis* Patton, 1905; *An. nili* Theobald, 1904; and *An. moucheti* Evans, 1925. Alongside these main vectors, several species of so-called “secondary vectors” contribute locally to continuous identified transmission of malaria [6–8]: *An. paludis* Theobald, 1900; *An. carnevalei* Brunhes et al., 1999 (see [9]); *An. coustani* Laveran, 1900; *An. marshallii* Theobald, 1903; *An. ziemanni* Gruenberg, 1902; *An. pharoensis* Theobald, 1901; *An. hancocki* Edwards, 1929; *An. wellcomei* Theobald, 1904; and *An. ovengensis* Awono-Ambene et al. 2004 (see [10]). The recent revision of the list of malaria vectors to include two additional species, *An. ziemanni* [11] and *An. rufipes* [12], suggests that full assessment of potential malaria vectors across the country is needed. Several studies conducted in areas where LLINs are used in large scale have revealed deltamethrin resistance in *An. gambiae* (*s.l*.) and *An. funestus* populations [13–16]. With the intensification of use of insecticidal vector control interventions, the landscape of local malaria epidemiology and insecticide susceptibility may change. New vectors may be introduced or well established vectors may become scarce, potentially with an expansion of insecticide resistance to marginalized potential vector species. In fact, changes in vectorial capacity may also occur in some anopheline species previously known as non-competent malaria vector species, such as *An. rufipes*.

*Anopheles rufipes* Gough, 1910, which belongs to the subgenus *Cellia* and series *Neocellia* is mostly distributed in tropical savannas of the sub-Saharan region. Apart from this typical form, there is a darkform (*Anopheles rufipes brousseri* Edwards, 1929) also found in these areas [17]. In Cameroon, the typical form *An. rufipes*, is regularly found in mosquito collections from northern region [12]. Its larvae normally develop in various standing and open water pools (e.g. rice fields, stream pools) which are also typical aquatic habitats for immature stages of *An. gambiae* (*s.l.*). In North Cameroon, main vector species belong to the *An. gambiae* complex, *An. funestus* (*s.s.*) and *An. pharoensis* [7], for which patterns of feeding and resting behavior, as well as the status of susceptibility to insecticides are increasingly documented [7, 13–16]. This region has a long history of pesticide utilization in agriculture and vectors have developed phenotypic resistance to DDT and pyrethroids, with multiple insecticide resistance mechanisms [14, 15, 18]. However, despite the abundance of *An. rufipes* in northern regions of Cameroon, very little attention has been given to this species bionomic as it was previously considered a non-competent malaria vector based on its zoophilic tendencies [17]. Following the recent publication of a cross-sectional survey on the role of this species in human malaria transmission in North Cameroon [12], we report further information on its feeding and resting habits, and first evaluation of its susceptibility to deltamethrin, the main pyrethroid insecticide of LLINs used in this area.

## Methods

### Study period and sites

**Fig. 1:**
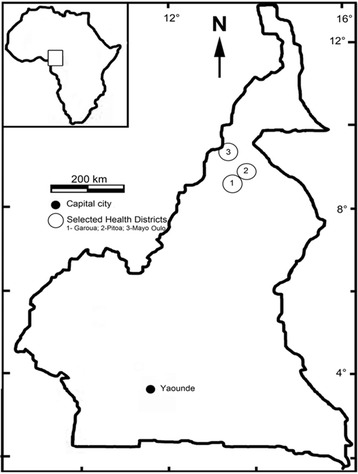
Map of Cameroon showing the location of the three selected health districts

Cross-sectional studies were conducted once each year during the rainy season (September to November) from 2011 to 2014 in 38 locations (clusters) belonging to three health districts (Garoua, Mayo Oulo and Pitoa) in the North region of Cameroon (Fig. 1). In the year 2015, the field survey was limited to larval collections and susceptibility tests on adults in different study sites. The three health districts are located in the tropical domain of the North region of Cameroon, and served as regional sentinel sites for monitoring of the efficacy of LLINs since the nationwide mass distribution of 2011. The full description of the selected health districts and their respective study sites has been made in previous reports [12, 19].

### Mosquito collections

Mosquito collections were performed every year between September and November using the dipping technique for larval collections and three conventional adult mosquito sampling methods: outdoor clay pots (OCPs), window exit traps (WETs) and indoor spraying collections (ISCs) [20, 21]. The adult mosquito trapping methods were chosen to specifically target resting mosquitoes as they enter or leave the houses, and also when they rest outdoors [22].

OCPs used as outdoor shelters for mosquitoes were approximately 0.5 m in diameter with an opening 20 cm wide. At each location, 9 OCPs were used for trapping outdoor resting mosquitoes in 3 dwellings each one separated by approximately 200–300 m. Per dwelling, a set of 3 OCPs were placed outside in a radius of 1–5 m from the houses, with the opening mouth directed away from sunlight. Five to ten liters of water were poured into each pot to keep it moist during the two consecutive nights of sampling. OCPs were placed at 18:00 h and left overnight. OCPs were then visited every morning between 7:00 and 8:00 h and mosquitoes found inside the pots were collected with mouth aspirators and transferred into paper cups for subsequent analyses.

WETs were set up from 18:00 to 7:00 h to collect mosquitoes that attempted to escape from bedrooms. Per location, 10 rooms were selected and equipped with WETs adapted from the model developed by Muirhead-Thomson [23, 24]. WETs were placed over the window of each selected bedroom and left overnight for 2 consecutive days. Mosquitoes were then collected from each trap every morning between 7:00 and 9:00 h using a mouth aspirator, and transferred in paper cups for further analyses.

ISCs were performed once between 6:00 and 9:00 h in rooms used for WETs. After covering the entire floor space and objects with white sheets, the rooms were then sprayed with commercial aerosols containing deltamethrin insecticide and closed for 10–15 min. Mosquitoes that fell on the sheets were picked up, counted and individually preserved on silica gel in tubes.

For larval collections, anopheline larvae and pupae samples were collected by dipping from active breeding sites [20]. Each year, samples were pooled per study site and brought to a local insectary rearing conditions, until F_0_ adult emergence.

### Mosquito processing

Adult specimens were morphologically identified using keys for the species of the genus *Anopheles* [17], and *An. rufipes* were separated from other local anopheline species by checking its typical characters on wings, legs and maxillary palp [25]. Members of the *An. gambiae* complex found in sympatry with *An. rufipes* were identified using PCR methods [26]. The physiological status of *Anopheles* samples was visually assessed as “blood-fed”, “gravid”, “half gravid” or “unfed”. All *Anopheles* specimens were screened for *P. falciparum* circumsporozoite protein (CSP) [27, 28] and for blood meal origin (if freshly fed *Anopheles* samples) by ELISA methods [29, 30]. For the latter, monoclonal antibodies against human, cow, pig, horse, chicken and sheep blood were used.

### Insecticide susceptibility testing

Susceptibility of adult *An. rufipes* mosquitoes to deltamethrin was assessed using WHO test kits and standard procedures [31]. Test kits including impregnated papers, test tubes and accessories were purchased from the WHO reference center at the Vector Control Research Unit, University Sains Malaysia. Insecticide susceptibility tests were performed on F_0_ females that emerged from aquatic stages (larvae and pupae). Batches of 20–25 two- to four-days-old unfed *An. rufipes* were exposed to filter papers impregnated with 0.05% deltamethrin. Another batch was at the same time exposed to untreated filter papers to serve as a control. The number of knocked down mosquitoes was recorded during exposure (60 min), and then tested mosquitoes were transferred to holding tubes with cotton pads soaked with 10% sugar to determine the mortality 24 h post-exposure. Susceptibility tests were concomitantly performed with the Kisumu susceptible reference strain of *An. gambiae* (*s.s.*).

### Data analysis

The circumsporozoite infection rate was calculated as the proportion of mosquitoes tested positive for *P. falciparum* circumsporozoite protein by ELISA. The overall human blood index (HBI) was determined as the proportion of mosquitoes identified to have fed on human blood by ELISA, i.e. included all mosquito samples positive for human blood meals either alone or mixed with other blood meals (undetermined blood sources were not considered). For each susceptibility test, the mortality rate was calculated as the proportion of dead mosquitoes over the total number of exposed specimens, when < 5% mortality was recorded in the control replicates. In the cases where the control mortality was ≥ 5% but < 20%, the mortality rate of tested samples was adjusted using Abbott’s formula [32]. Resistance status was evaluated according to the WHO criteria [31]. Knockdown times for 50 and 95% (KDT_50_ and KDT_95_) *An. rufipes* tested mosquitoes were estimated using a log probit model performed with WINDL software (version 2.0, 1999). The recorded KDT_50_ were compared with that of the Kisumu reference susceptible strain by estimates of KDT_50_ Ratios (KDT_50_R). For the statistical analysis, data were analyzed using Chi-square tests of the free online statistic tools of AnaStats 2016. The level of significance was α = 0.05.

## Results

### Anopheline density and distribution

**Fig. 2:**
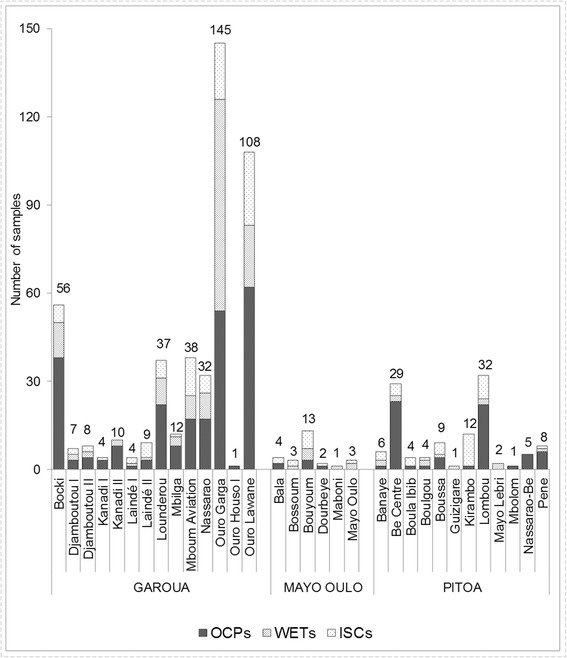
Overall number of adult *Anopheles rufipes* mosquitoes collected using three sampling methods in 32 study locations of North Cameroon

A total of 9327 adult *Anopheles* were collected during 4 successive years (2011–2014) using the three sampling methods, among which 609 *An. rufipes* individuals were identified (6.5%) alongside *An. gambiae* (*s.l.*) (71.8%) and *An. funestus* (*s.l.*) (20.8%) (Table 1). Six other species represented less than 1% (81/9327) of total samples i.e. *An. pharoensis*, *An. paludis*, *An. ziemanni*, *An. coustani*, *An. nili* and *An. longipalpis*. *Anopheles rufipes* samples was identified across 35 of 38 selected study locations (including 3 locations positive at larval stages) (Fig. 2).

**Table 1:** Number (*n*) and frequency (%) of *Anopheles rufipes* and other anophelines collected by three conventional methods from study districts of the North Cameroon

Sampling methods	*An. rufipes*		*An. gambiae* (*s.l*.)		*An. funestus*		*An. pharoensis*		*Anopheles* sp.		Total anophelines	
	*n*	%	*n*	%	*n*	%	*n*	%	*n*	%	*n*	%
OCP	312	3.35	1787	19.16	1231	13.20	11	0.12	13	0.14	3354	35.96
WETs	163	1.75	2400	25.73	174	1.87	21	0.23	10	0.11	2768	29.68
ISCs	134	1.44	2509	26.90	536	5.75	17	0.18	9	0.10	3205	34.36
Total/species	609	6.53	6696	71.79	1941	20.81	49	0.53	32	0.34	9327	100

### Resting behavior

The proportion of *An. rufipes* samples resting in outdoor pots was 51.2 *vs* 22.0% of those entering houses before resting indoors (*χ*^2^ = 70.244, *df* = 1, *P* < 0.001). As shown in Table 1 and compared with main vector species, *An. rufipes* [as *An. funestus* (*s.l.*)] showed a consistent propensity to rest outdoors than *An. gambiae* (*s.l.*) (*χ*^2^ = 130.561, *df* = 1, *P* < 0.001). This observation was enhanced by the high percentage of *An. rufipes* samples attempting to escape through WET (54.9%) after entering houses compared with that of *An. funestus* (*χ*^2^ = 85.412, *df* = 1, *P* < 0.001).

### Blood-feeding status and indices

In total 581 *An. rufipes* samples were checked for their feeding status, among which 379 (65.2%) were blood-fed, 125 (21.5%) unfed and 77 (13.3%) gravid and/or half-gravid. The highest percentage of blood-fed samples was recorded in WET (74.9%), followed by those collected by ISCs (64.0%) and in oudoor pots (60.5%), respectively This seems to be positively correlated with the exophilic habits of this vector species.

**Fig. 3:**
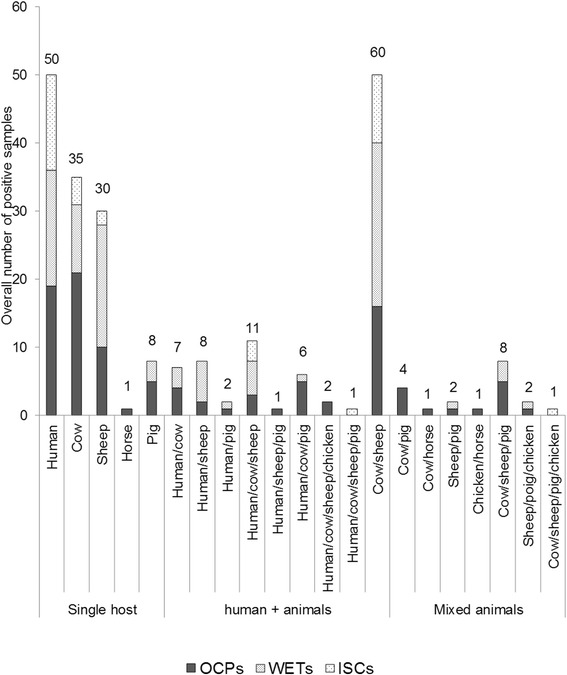
Blood meal composition of *Anopheles rufipes* collected in 2012 and 2013 in selected health districts of North Cameroon

A total of 329 blood samples from *An. rufipes* were checked for blood meal origins, and 22 different blood meal combinations were recorded. About 18.5% (*n* = 61) blood meals were exclusively from human origin, 12.8% (*n* = 42) were a mixture of human and animals, 30.1% (*n* = 99) were from five single animal hosts including cows (14.3%), sheep (11.6%), pigs (4%), chickens and horses, 24.3% (*n* = 80) were a mixture blood meals taken from two or more animal hosts and 14.3% (*n* = 47) were undetermined (Fig. 3).

The overall human blood index of *An. rufipes* was 37% with insignificant variations between indoor (53%), outdoor (35%) and window exiting (33%) samples (*χ*^2^ = 4.801, *df* = 2, *P* = 0.0907) (Table 2). This suggests plasticity in resting and feeding behavior developed by *An. rufipes* populations from study locations. As shown in Table 2, blood indices of each animal host also displayed variations either by vector species or by resting places.

**Table 2:** Overall human and animal blood indices of *Anopheles rufipes* and main anopheline species from 2011 to 2014 in North Cameroon

Species	Method	Human	Cow	Sheep	Pig	Chicken	Horse
*An. rufipes*	OCP (*n* = 143)	0.35	0.55	0.41	0.20	0.03	0.02
	WET (*n* = 103)	0.33	0.50	0.60	0.15	0.01	0.00
	ISC (*n* = 36)	0.53	0.53	0.47	0.06	0.03	0.00
	Total (*n* = 282)	0.37	0.52	0.49	0.16	0.02	0.01
*An. gambiae* (*s.l.*)	OCP (*n* = 236)	0.62	0.40	0.40	0.12	0.03	0.00
	WET (*n* = 159)	0.85	0.22	0.16	0.09	0.04	0.00
	ISC (*n* = 689)	0.69	0.30	0.25	0.10	0.03	0.01
	Total (*n* = 1084)	0.70	0.31	0.27	0.11	0.03	0.01
*An. funestus*	OCP (*n* = 240)	0.35	0.54	0.57	0.19	0.01	0.00
	WET (*n* = 25)	0.36	0.64	0.40	0.04	0.08	0.08
	ISC (*n* = 165)	0.25	0.44	0.69	0.16	0.01	0.02
	Total (*n* = 430)	0.31	0.51	0.60	0.17	0.01	0.01

*Abbreviation*: *n* number tested positive (undetermined not included)

### Malaria parasite infection

The ELISA screening for the presence of *Plasmodium* circumsporozoite protein found two positive individuals (0.39%) among 517 *An. rufipes* tested. This species contributed for less than 2% of the global CSP infection rate of 1.26% (± 0.23%), dominated by *An. gambiae* (*s.l.*) (~ 90%) (Table 3).

**Table 3:** Rate of *Plasmodium falciparum* circumsporozoite protein positivity (CSP+) of *Anopheles rufipes* and other malaria vector species by sampling methods from 2011 to 2014 in North Cameroon

Sampling method	*An. rufipes*			*An. gambiae* (*s.l.*)			*An. funestus*		
	Tested	CSP+	% (95% CI)	Tested	CSP+	% (95% CI)	Tested	CSP+	% (95% CI)
OCP	258	1	0.39 (0.34–0.44)	1773	18	1.02 (1.01–1.03)	1223	3	0.25 (0.24–0.26)
WETs	143	1	0.70 (0.59–0.81)	2386	26	1.09 (1.08–1.10)	169	0	0
ISCs	116	0	–	2466	59	2.39 (2.38–2.40)	528	5	0.95 (0.91–0.99)
Total	517	2	0.39 (0.37–0.41)	6625	103	1.56 (1.55–1,57)	1920	8	0.42 (0.41–0.43)

*Abbreviation*: *CI* confidence interval

### Status of susceptibility to deltamethrin

**Fig. 4:**
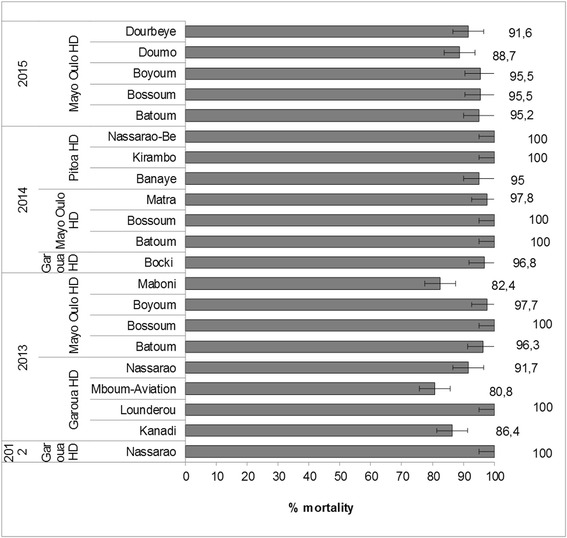
Mortality rates (%) following exposure to 0.05% deltamethrin impregnated papers of *Anopheles rufipes* populations from Garoua, Pitoa and Mayo Oulo Health Districts collected between 2012 and 2015

Between 2012 and 2015, 21 bioassays were performed (Table 4). In all, susceptibility tests were performed on a total of 1092 female *An. rufipes* representing 59, 198 and 835 samples from Pitoa, Garoua and Mayo Oulo health districts, respectively. The recorded KDT_50_, KDT_95_ and KDT_50_R are presented in Table 4 and the mortality rates in Fig. 4.

**Table 4:** Knockdown times (KDT_50_ and KDT_95_) and KDT_50_ ratios (KDT_50_R), following exposure to 0.05% deltamethrin of *Anopheles rufipes* (*s.l.*) populations from study locations of North Cameroon from 2012 to 2015

Year	Health district	Location	*n*	KDT_50_ (95%CI) (min)	KDT_95_ (95%CI) (min)	KDT_50_ ratio	Status
2012	Garoua	NAS	48	17.5 (15.4–19.4)	42.0 (36.5–51.0)	1.84	S
2013	Garoua	Kanadi	22	22.6 (20.3–25.0)	37.2 (34.2–46.5)	2.38	R
		Lounderou	28	21.6 (19.1–24.0)	40.2 (34.9–47.9)	2.27	S
		Mboum Aviation	26	35.2 (31.7–38.6)	70.2 (60.6–87.9)	3.71	R
		Nassarao	12	24.6 (21.2–28.2)	39.8 (33.6–54.7)	2.59	SR
	Mayo Oulo	Batoum	80	21.3 (19.3–23.2)	54.5 (48.3–63.7)	2.24	SR
		Bossoum	32	21.5 (18.2–24.5)	53.0 (44.5–69.1)	2.26	S
		Boyoum	87	25.0 (23.4–26.6)	55.9 (50.6–63.4)	2.63	SR
		Maboni	51	32.4 (29.7–35.5)	80.2 (68.2–100.4)	3.41	R
2014	Garoua	Bocki	62	21.9 (20.1–23.7)	48.3 (43.0–56.2)	2.31	SR
	Mayo Oulo	Batoum	107	9.9 (9.0–10.7)	27.0 (24.1–30.9)	1.04	S
		Bossoum	43	18.0 (15.2–20.5)	39.4 (34.0–48.7)	1.90	S
		Matra	90	21.2 (19.8–22.7)	51.3 (46.2–58.4)	2.23	SR
	Pitoa	Banaye	20	13.9 (8.3–18.1)	39.7 (31.2–61.0)	1.46	SR
		Kirambo	26	10.5 (4.6–15.7)	41.5 (30.8–63.2)	1.11	S
		Nassarao-Be	13	5.4 (0.8–10.9)	46.1 (25.5–174.8)	0.57	S
2015	Mayo Oulo	Batoum	21	27.5 (24.1–31.2)	60.1 (49.9–80.5)	2.90	SR
		Bossoum	67	23.7 (22.1–25.4)	49.1 (44.3–56.1)	2.50	SR
		Boyoum	89	28.0 (26.2–29.8)	65.1 (58.4–74.5)	2.95	SR
		Doumo	97	26.6 (21.4–23.8)	45.4 (41.7–50.3)	2.80	R
		Dourbeye	71	30.7 (28.3–33.0)	68.7 (60.8–80.8)	3.23	SR

*Abbreviations*: *n* number tested, *CI* confidence interval, *S* susceptible, *SR* suspected resistance to be confirmed, *R* resistance

Seven *An. rufipes* populations from six locations (Nassarao, Lounderou, Batoum, Bossoum, Kirambo and Nassarao-Be) revealed susceptible to deltamethrin (100% mortality) with KDT_50_ and KDT_95_ ranges of 5.4–21.6 and 40.0–46.1 min respectively (0.57 ≤ KDT_50_R ≤ 2.27). Four *An. rufipes* populations in 4 different locations (Kanadi, Maboni, Doumo and Mboum aviation) showed resistance to deltamethrin (mortality from 80.8 to 88.7%), with KDT_50_ and KDT_95_ ranges of 22.6–35.2 and 37.2–80.2 min, respectively (2.38 ≤ KDT_50_R ≤ 3.71). Finally, 10 *An. rufipes* populations from 8 clusters (Nassarao, Batoum, Boyoum, Bocki, Matra, Banaye, Bossoum and Dourbeye) showed probable resistance (from 91.6 to 97.8% mortality) with KDT_50_ and KDT_95_ ranges of 13.9–30.7 and 39.7–68.7 min, respectively (1.46 ≤ KDT_50_R ≤ 3.23).

The trends in mortality rates (± standard deviation) of *An. rufipes* showed unpredictable variations across locations and years of collection. Deltamethrin susceptibility among populations was more distributed in 2014, with four “susceptible” populations and three remaining populations classified “probably resistant”, compared with year 2013 with two susceptible populations, three ranged as “probably resistant” and three as “resistant”, and year 2015 with no susceptible population out of five tested. In addition, *An. rufipes* populations from Nassarao (2012 and 2013), Bossoum and Batoum (2013, 2014 and 2015) displayed changes on their status from “susceptible” one year to “probably resistant” another year and *vice versa*.

## Discussion

The present paper is complimentary to a recently published paper which highlighted for the first time in Cameroon the important epidemiological role of *An. rufipes* in malaria transmission, with 0–0.481 infectious bites/person/night recorded in study locations [12]. Few reports from western and southern Africa [32–37] are in accordance with this recent studies conducted so far in Cameroon*.*

The study objective was to present additional information on *An. rufipes* populations from the North Cameroon with a focus on its resting and feeding behaviors as well as on its susceptibility to deltamethrin insecticide after the nationwide distribution of LLINs in Cameroon in 2011. From the study, it appeared that *An. rufipes* was widely distributed in both suburban and rural locations in the study area, concomitant with known major malaria vector species of the *An. gambiae* complex (*An. arabiensis*, *An. gambiae* and *An. coluzzii*), *An. funestus* (*s.s.*) and *An. pharoensis*. This composition of resting individuals of malaria vectors has been frequently reported from savanna villages in Cameroon [7, 38] and elsewhere in Africa [36, 37, 39, 40]. This composition of resting vector populations does not necessarily reflect the relative abundance of a given species in the field; species including *An. rufipes* have displayed high and significant numbers of resting samples compared with that collected on human volunteers in same location in Senegal [39] and Chad [40]. Normally, *Anopheles rufipes* breed in various standing water bodies including marshes, pools, rice fields, river banks, temporary streams locally called “Mayos” which are also prolific, most especially for *An.gambiae* (*s.l.*) larval development.

*An. rufipes* was found to be highly opportunistic regarding its feeding and resting habits; this species was found to feed on a large variety of hosts including human, cows, sheep, pigs, chickens, horses and potentially other undetermined animals [12]. This observation was confirmed by our findings showing that, in the presence of alternative hosts, *An. rufipes* were less anthrophagic than zoophagic, about 37% of it blood meals were from human while up to 50% were from animals (*P* < 0.001). However, the preferred animal hosts were cows, sheep and pigs. This zoophagic propensity of *An. rufipes* is not uncommon and has been reported previously in the field [17]. Meanwhile, the combination of various blood meal origins, including human and animal hosts, is not unusual and has also been reported in well-known malaria vector species such as members of the *An. gambiae* complex, *An. funestus* and *An. pharoensis* in Cameroon [19, 38] and other tropical African countries [41–43].

Concerning the resting behavior, the sampling methods used have been previously applied as standards to sample mosquitoes resting around and inside human dwellings [44–47]. Based on this distribution in sampling methods, *An. rufipes* exhibited endophilic and exophilic behaviour in the study sites, consistent with flexibility observed in the local malaria vectors species *An. arabiensis*, *An. funestus* and *An. pharoensis*. The best example of this behavioral plasticity is the widespread African malaria vector species, *Anopheles arabiensis*, which is capable of adapting its feeding responses according to various situations by feeding on human outdoors or on alternative animal hosts [48]. Any species with such behavioural heterogeneity in the field should be regarded as a potential target for the control of residual malaria parasite transmission [49].

More importantly, two *An. rufipes* specimens were found positive for CSP, one from Ouro Lawane in 2013 (*n* = 38) and one from Lombou in 2014 (*n* = 17). This finding confirms the ability of this species to carry human malaria parasites, as it has been also demonstrated in a parallel study on transmission profiles carried out in the same locations [12]. This further observation is remarkable since several authors ranked this species as zoophilic, with very little or no epidemiological importance in some areas. The screening of the presence of malaria infection in mosquitoes was continuously improved since the first evidence of sporozoites in a single *An. rufipes* specimen 60 years ago to date with the detection of parasite antigens by ELISA and PCR methods. These advanced techniques frequently detect as positive for *Plasmodium* sporozoites several species that are not considered vectors, and provide little indication of the transmission ability of such species [50–54]. Based on these observations, *An. rufipes* could henceforth be considered as a potential vector in North Cameroon, indicating that it should also be considered during monitoring along with other malaria vector species of the vectorial system.

Furthermore, by assessing for the first time the susceptibility of *An. rufipes* populations to deltamethrin, one of the common pyrethroid insecticides used in LLINs distributed nationwide in 2011 and in 2016 in Cameroon, we observed that mortality rates were highly variable depending on the location and time point. The distribution of confirmed or suspected resistance in 12 of the 15 tested *An. rufipes* populations is indicative of the development of phenotypic resistance to pyrethroids in the three surveyed health districts. In addition to previous reports on pyrethroid resistance in members of the *An. gambiae* complex from the same locations [19, 55, 56], these data highlight the extent of insecticide resistance in potential malaria vector populations from North Cameroon. This is the first report of pyrethroid resistance in *An. rufipes* from Cameroon, which may have a potential impact on the efficacy of LLINs in study health districts. Insecticide resistance in *An. rufipes* populations and other vector species of *An. gambiae* complex and *An. funestus* group from this region should therefore be hence monitored according to the new WHO guidelines [57], in order to guide comprehensive and data-driven planning and implementation of vector control.

## Conclusions

The current study gathered relevant information on the resting and feeding behavior and deltamethrin susceptibility of *An. rufipes* populations from North Cameroon relative to other malaria vectors, and confirmed that this species may have a potential role in local malaria epidemiology. The findings indicate that *An. rufipes* should be considered in monitoring programs for malaria vectors in North Cameroon, and potentially throughout the tropical domain of African countries.

## Abbreviations

CSP: circumsporozoite protein
ELISA: enzyme-linked immunosorbent assay
ISCs: indoor spraying catches
KDT: knockdown times
LLINs: long-lasting insecticidal treated nets
OCPs: outdoor clay pots
WETs: window exit traps
WHO: World Health Organization
